# Male-biased protein expression in primordial germ cells, identified through a comparative study of UAS vectors in *Drosophila*

**DOI:** 10.1038/s41598-021-00729-1

**Published:** 2021-11-02

**Authors:** Masaki Masukawa, Yuki Ishizaki, Hiroki Miura, Makoto Hayashi, Ryoma Ota, Satoru Kobayashi

**Affiliations:** 1 Degree Programs in Life and Earth Sciences, Graduate School of Science and Technology, University of Tsukuba, Tsukuba, Ibaraki 305-8577 Japan; 2 Life Science Center for Survival Dynamics, Tsukuba Advanced Research Alliance (TARA), University of Tsukuba, Tsukuba, Ibaraki 305-8577 Japan; 3 Graduate School of Life and Environmental Sciences, University of Tsukuba, Tsukuba, Ibaraki 305-8577 Japan; 4 Department of Biosciences, Faculty of Science and Engineering, Teikyo University, Utsunomiya, Tochigi, 320-8551 Japan; 5 Division of Integrated Science and Engineering, Graduate School of Science and Engineering, Teikyo University, Utsunomiya, Tochigi, 320-8551 Japan

**Keywords:** Cell biology, Developmental biology

## Abstract

In *Drosophila*, three types of UAS vectors (UASt, UASp, and UASz) are currently available for use with the Gal4-UAS system. They have been used successfully in somatic cells and germline cells from ovaries. However, it remains unclear whether they are functional in the germline cells of embryos, larvae, and adult testes. In this study, we found that all three types of UAS vectors were functional in the germline cells of embryos and larvae and that the UASt and UASz vectors were active in the germline of the distal tip region in adult testes. Moreover, we observed that protein expression from the UAS vectors was male-biased in germline cells of late embryos, whereas their respective mRNA expression levels were not. Furthermore, O-propargyl-puromycin (OPP) staining revealed that protein synthesis was male-biased in these germline cells. In addition, GO terms related to translation and ribosomal maturation were significantly enriched in the male germline. These observations show that translational activity is higher in male than in female germline cells. Therefore, we propose that male-biased protein synthesis may be responsible for the sex differences observed in the early germline.

## Introduction

The Gal4-UAS system is a tool widely used to induce gene expression in *Drosophila*. This system is composed of two factors: an upstream activation sequence (UAS) and the yeast-derived Gal4 protein, which binds to the UAS sequence and activates downstream gene expression^1^.

**Figure 1:**
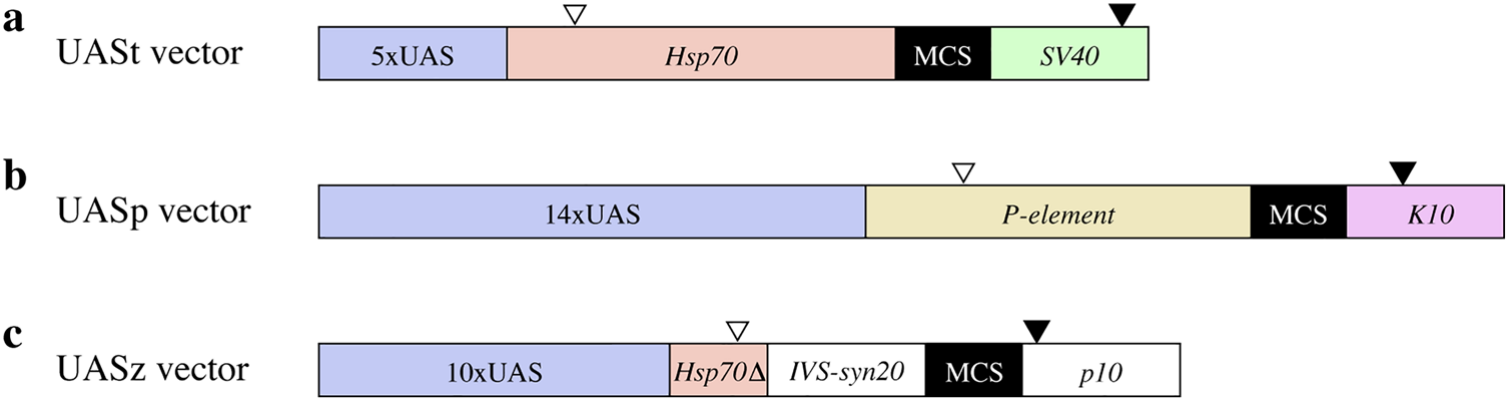
Three types of UAS vectors. Schematic diagrams for the (**a**) UASt, (**b**) UASp, and (**c**) UASz vectors. The UASt vector contains a 5xUAS, a *Hsp70*-derived core promoter with a 203-bp 5´UTR, a multiple cloning site (MCS) containing a *Kpn*I site, and a *SV40* terminator. The UASp vector contains a 14xUAS, a *P*-*element*-derived core promoter with a 194-bp 5´UTR, a MCS, and a *K10* terminator. The UASz vector contains a 10xUAS, a *Hsp70*-derived core promoter with a 19-bp 5´UTR, in which the sequences targeted by *Hsp70* piRNAs were deleted, a myosin IV intron and synthetic UTR elements (*IVS-syn21*), a MCS, and a *p10* terminator. White triangles indicate transcription start sites. Black triangles indicate poly(A) addition sites.

Three types of UAS vectors are currently available for use in *Drosophila*. The first vector developed, UASt, contains a *Hsp70-*derived core promoter downstream of the UAS sequence and a *simian virus 40* (*SV40*) terminator^2–4^ (Fig. 1a). Although the UASt vector is active in somatic tissues, UASt-driven gene expression in the germline cells from adult ovaries is poor^5, 6^. To overcome this issue, the UASp vector is used to induce gene expression in germline cells^5^. The UASp vector contains a *P-element*-derived core promoter and a *K10* terminator^5^ (Fig. 1b). Poor protein expression observed when using the UASt vector is caused by the *Hsp70*-derived core promoter, which contains a short *Hsp70* 5´UTR sequence that is targeted by Piwi-interacting RNAs (piRNAs)^6, 7^. Therefore, the sequence targeted by the *Hsp70* piRNAs was deleted from UASt to generate the UASz vector^6^ (Fig. 1c). The UASz vector is active in the germline cells from adult ovaries and in somatic tissues, and gene expression is higher than observed with UASp^6^.

Although protein expression from these three UAS vectors has been examine in the germline cells from adult ovaries, it remains unclear whether they are active in the germline cells from embryos, larvae, and adult testes. Therefore, we examined gene expression from these UAS vectors under the control of the *nos-Gal4* driver, which is active in a germline-specific manner^8^, in both the male and female germline cells from embryos, larvae, and adults.

During the course of this study, we discovered that protein expression from all of the UAS vectors was male-biased in the germline cells from late embryos. We further found that OPP staining, a sensitive method for detecting protein synthesis, was also male-biased in these germline cells. In addition, GO terms related to translation and ribosomal maturation were significantly enriched in the male germline. Therefore, we conclude that translational activity is higher in male than in female germline cells. Sex-biased gene expression was identified in the somatic cells of embryos^9, 10^ and adults^9, 11^ and in germline cells during embryogenesis^12^ and gametogenesis^13, 14^ using RNA-sequencing and microarray analyses. Furthermore, genes involved in sex determination of the somatic and germline cells were identified^15, 16^. However, no reports describe overall male-biased translation in either germline or somatic cells, although translation of *male-specific lethal 2* (*msl2*) mRNA is specifically repressed by the upstream regulator protein Sex lethal (Sxl) in the female soma^17^.

## Materials and methods

### Fly stocks

Flies were maintained on standard *Drosophila* medium at 25 °C. Fly strains used in this study were as follows: *y w, vasa-EGFP*^18^, *nanos-Gal4-VP16* (*nos-Gal4*)^8^, and *UASp-EGFP-K10 3´UTR* (*UASp-EGFP*). The *UASp-EGFP* transgene is in the same attP site as the *UASt-* and *UASz-EGFP* transgenes^19^. *UASt-Redstinger* (*UASt-RFP* I) (stock No. 8545), *w*^*1118*^; *UASt-Redstinger* (*UASt-RFP* III) (stock No. 8547), and *y*^*1*^ M{vas-int.Dm}ZH-2A w*; *M{3xP3-RFP.attP´}ZH86Fa* (stock No. 24486) were obtained from the Bloomington Drosophila Stock Center.

### Production of UASt- and UASz-EGFP flies

To produce fly strains carrying *UASt-* and *UASz-EGFP*, the *EGFP* coding region was amplified from pEGFP-N1 (Clontech) using the primer pairs UASt-KpnI-EGFP-Fw/UASt-KpnI-EGFP-Rv and UASz-KpnI-EGFP-Fw/UASz-KpnI-EGFP-Rv, respectively (Table S1). The amplified DNA fragments were cloned into *Kpn*I-digested pUASt-attB^20^ and *Kpn*I-digested pUASz-1.0^6^, respectively, using the In-Fusion HD Cloning Kit (Takara Bio., Cat. No. Z9648N). The resultant vectors were injected into *y*^*1*^ M{vas-int.Dm}ZH-2A w*; *M{3xP3-RFP.attP´}ZH86Fa* embryos to produce flies carrying *UASt-* and *UASz-EGFP* in the attP site on the third chromosome.

### Fixation of embryos and larval and adult gonads

Embryos were derived from females homozygous for *nos-Gal4* mated with males homozygous for *UASt-*, *UASp-*, or *UASz-EGFP* or *UASt-RFP* III. Larvae and adults were derived from females homozygous for *nos-Gal4* mated with males homozygous for *UASt-*, *UASp-*, or *UASz-EGFP*. The embryos were dechorionated in a sodium hypochlorite solution. The dechorionated embryos were fixed in 1:1 heptane:fixative [4% paraformaldehyde in PBS (130 mM NaCl, 7 mM Na_2_HPO_4_, and 3 mM NaH_2_PO_4_)] for 30 min. Vitelline membranes were removed by vigorously shaking the embryos in 1:1 methanol:heptane. The embryos were then rinsed with methanol and stored in methanol at − 20 °C.

Larval gonads were dissected from first-, second-, and third-instar larvae and fixed for 15 min. The fixed gonads were rinsed with PBSTw (PBS containing 0.2% Tween 20), rinsed with methanol, and stored in methanol at − 20 °C.

Adult gonads were dissected from flies 4–6 days after eclosion and were fixed for 15 min. The fixed testes and ovaries were rinsed with PBSTw and stored at 4 °C.

### Immunostaining

Immunofluorescence staining of embryos was performed as described^21^. Briefly, the fixed embryos were incubated with 3:1, 2:2, and 1:3 methanol:PBSTr (PBS containing 0.1% Triton X-100) for 3 min each and washed with PBSTr three times for 15 min each. After washing, the embryos were incubated with blocking solution (PBS containing 2% BSA, 0.1% Tween 20, and 0.1% Triton X-100) for 1 h. After blocking, the embryos were incubated overnight at 4 °C with blocking solution containing primary antibodies. The embryos were washed with PBSTr three times for 15 min each and incubated overnight at 4 °C with the blocking solution containing secondary antibodies. The primary antibodies used were as follows: rabbit anti-EGFP (1:500; Thermo Fisher Scientific, Cat. No. A11122), rabbit anti-DsRed (1:1000; Takara, Cat. No. Z2496N), chick anti-Vasa (1:2000)^22^, and mouse anti-Sxl (1:20; Developmental Studies Hybridoma Bank (DSHB), M18). The secondary antibodies used were as follows: Alexa Fluor 488–conjugated goat anti-rabbit (1:500; Thermo Fisher Scientific, Cat. No. A11034), Alexa Fluor 546–conjugated goat anti-rabbit (1:500; Thermo Fisher Scientific, Cat. No. A11035), Alexa Fluor 633–conjugated goat anti-chick (1:500; Thermo Fisher Scientific, Cat. No. A21103), Alexa Fluor 488–conjugated goat anti-mouse (1:500; Thermo Fisher Scientific, Cat. No. A11029), and Alexa Fluor 546–conjugated goat anti-mouse (1:500; Thermo Fisher Scientific, Cat. No. A11030). The embryos were washed with PBSTr three times for 15 min each and mounted in VECTASHIELD Mounting Medium (VECTOR, Cat. No. H-1000). The sex of each embryo was determined using Sxl staining (Sxl is expressed in the soma in a female-specific manner).

Immunofluorescence staining of larval and adult gonads was performed as described^23^. Briefly, the fixed gonads were washed with PBSTr three times for 15 min each. The gonads were then incubated with blocking solution for 1 h. After blocking, the gonads were incubated overnight at 4 °C with blocking solution containing the following primary antibodies: rabbit anti-EGFP (1:500), chick anti-Vasa (1:2000), and mouse anti-Fas3 (1:20; DSHB, 7G10). The gonads were washed with PBSTr three times for 15 min each and incubated overnight at 4 °C with blocking solution containing the following secondary antibodies: Alexa Fluor 488–conjugated goat anti-rabbit (1:500), Alexa Fluor 633–conjugated goat anti-chick (1:500), and Alexa Fluor 546–conjugated goat anti-mouse (1:500). The gonads were washed with PBSTr three times for 15 min each and mounted in VECTASHIELD Mounting Medium. Sexing of larval gonads was performed using Fas3 staining.

**Figure 2:**
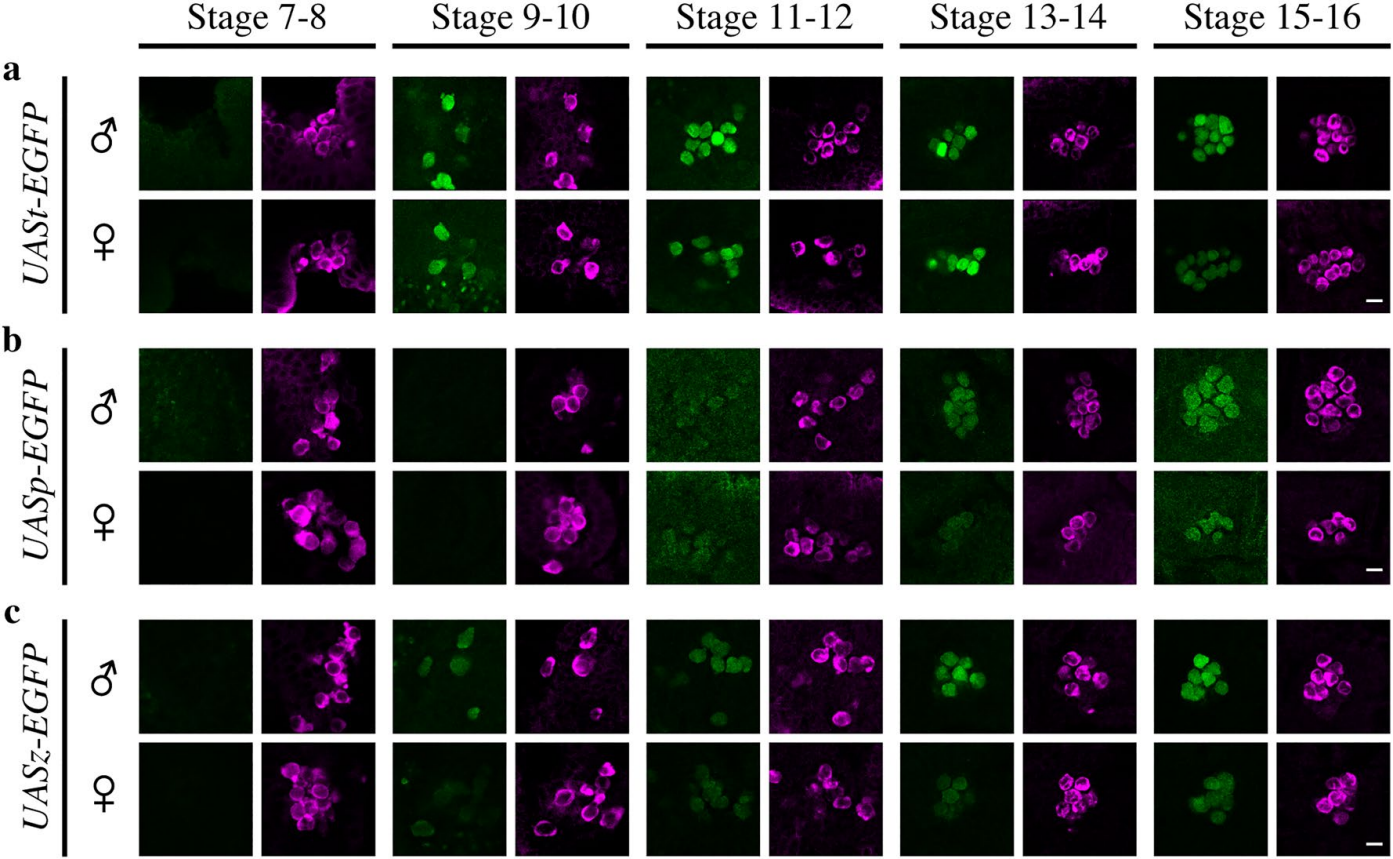
EGFP expression from *UASt*-, *UASp*-, and *UASz-EGFP* in PGCs. EGFP expression from (**a**) *UASt-EGFP*, (**b**) *UASp-EGFP*, and (**c**) *UASz-EGFP* in male (♂, upper panels) and female (♀, lower panels) PGCs from embryos at stages 7–8, 9–10, 11–12, 13–14, and 15–16. EGFP (green, left) and Vasa (magenta, right) fluorescence are shown. Embryos, derived from females homozygous for *nos-Gal4* mated with *UAS-EGFP* homozygous males, were immunostained for EGFP (green), Vasa (magenta, a marker for PGCs), and Sxl (not shown, used to sex the PGCs). Signal was obtained using a confocal laser fluorescence microscope with laser intensities and detector settings appropriate for the expression level of EGFP from each *UAS-EGFP* construct at each stage. However, the conditions for signal acquisition were identical for male and female PGCs at each stage. Scale bars: 10 µm.

**Figure 3:**
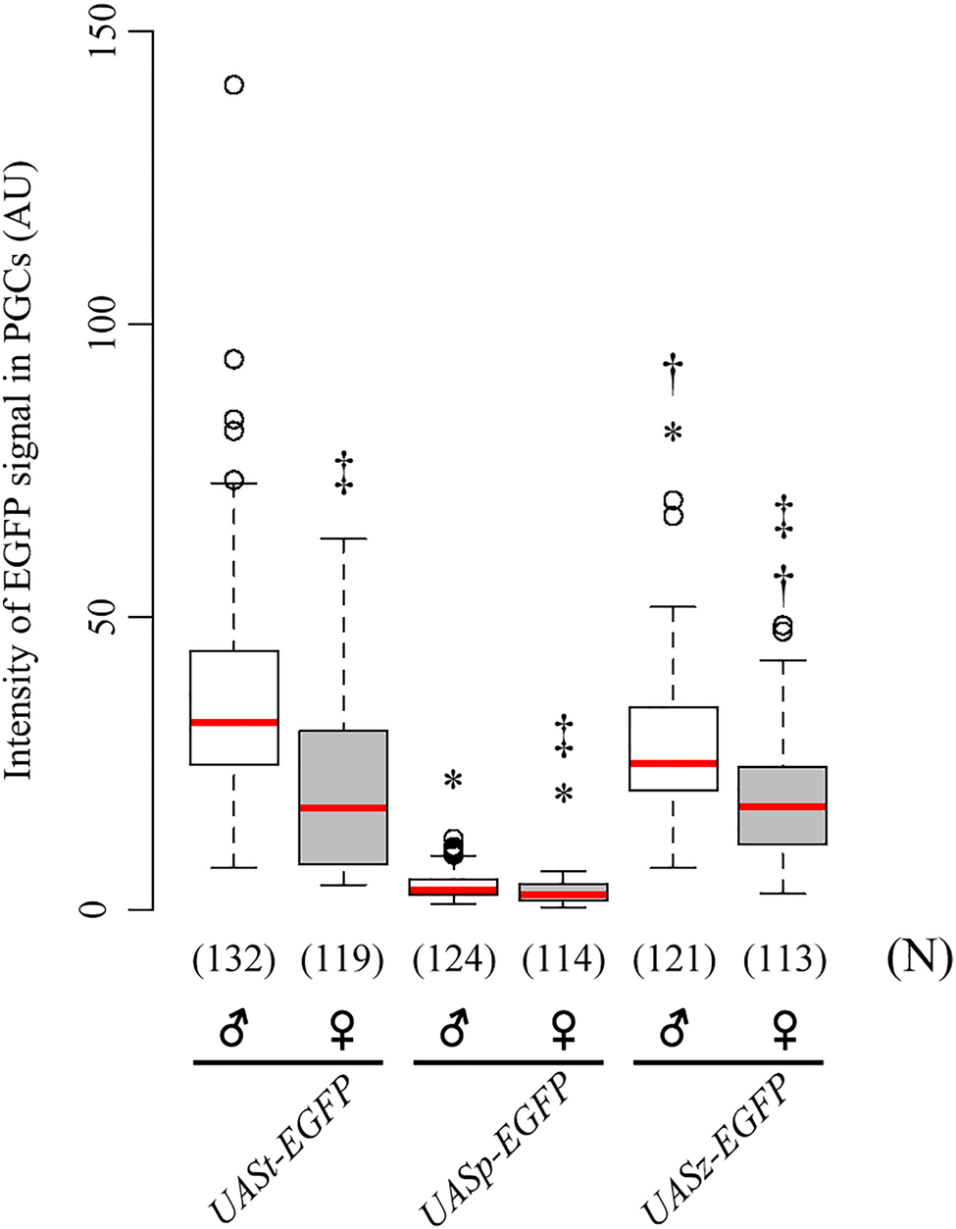
Quantification of EGFP expressed from *UASt*-, *UASp*-, and *UASz-EGFP* in PGCs. The level of EGFP expressed from *UASt-EGFP*, *UASp-EGFP*, and *UASz-EGFP* in male (♂, white) and female PGCs (♀, grey) from stage-15–16 embryos. The embryos were obtained as stated in Fig. 2. The pixel intensity for the EGFP signal observed is shown. Pixel intensities were obtained using a confocal laser fluorescence microscope with fixed laser intensities and detector settings regardless of the expression level of EGFP from each UAS vector. ﻿﻿Each box plot represents median values (red bars) and first (25%) and third (75%) quartile values. Whiskers extend 1.5 times the interquartile range (IQR) from the 25% and 75% quartile. The upper and lower whisker indicate the largest and smallest value that are no greater and lower than 75% plus 1.5 IQR and 25% minus 1.5 IQR, respectively. White circles represent outliers. Significance was calculated using the Mann–Whitney *U* test. *P < 0.01, *UASt-EGFP* vs. *UASp-EGFP* or *UASz-EGFP*. †P < 0.01, *UASp-EGFP* vs. *UASz-EGFP*. ‡P < 0.01, male vs. female PGCs. The number of PGCs (N) examined is indicated in parentheses. AU: arbitrary units.

**Figure 4:**
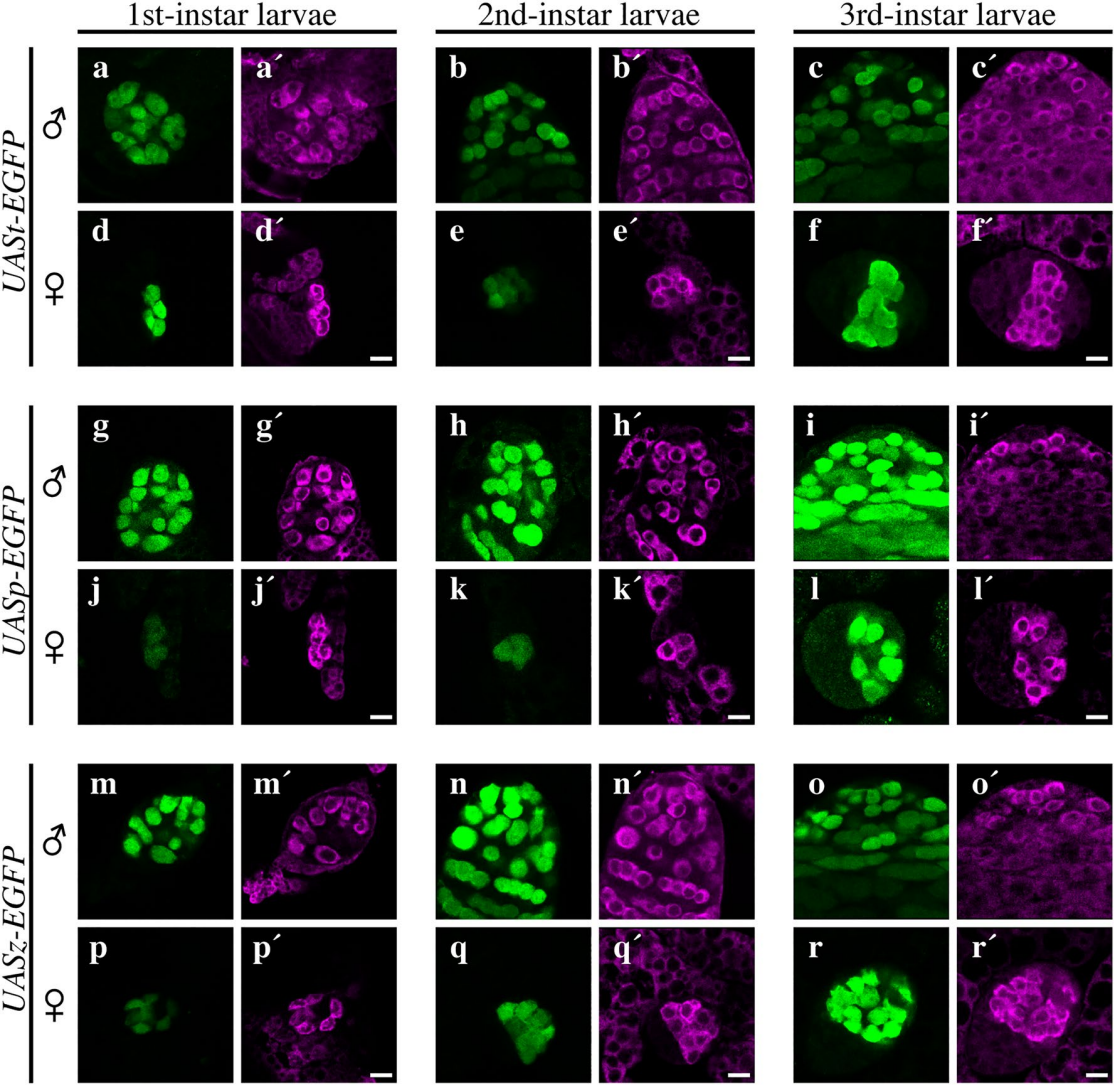
Expression of EGFP from *UASt-*, *UASp-*, and *UASz-EGFP* in the male and female germline cells from larvae. EGFP expression from (**a**–**f**) *UASt-*, (**g**–**l**) *UASp-*, and (**m**–**r**) *UASz-EGFP* in male (**a**–**c**, **g**–**i**, and **m**–**o**) and female (**d**–**f**, **j**–**l**, and **p**–**r**) germline cells from first- (**a,d,g,j,m,p**), second- (**b,e,h,k,n,q**), and third-instar (**c,f,i,l,o,r**) larvae. Vasa fluorescence (magenta) indicates germline cells. Gonads were dissected from larvae derived from females homozygous for *nos-Gal4* mated with *UAS-EGFP* homozygous males. The gonads were then immunostained for EGFP (green), Vasa (magenta), and Fas3 (not shown, a marker for Hub cells observed only in males). Signal was obtained using a confocal laser fluorescence microscope with laser intensities and detector settings appropriate for the expression level of EGFP from each *UAS-EGFP* construct at each stage. However, the conditions for signal acquisition were identical for male and female PGCs at each stage. Scale bars: 10 µm.

**Figure 5:**
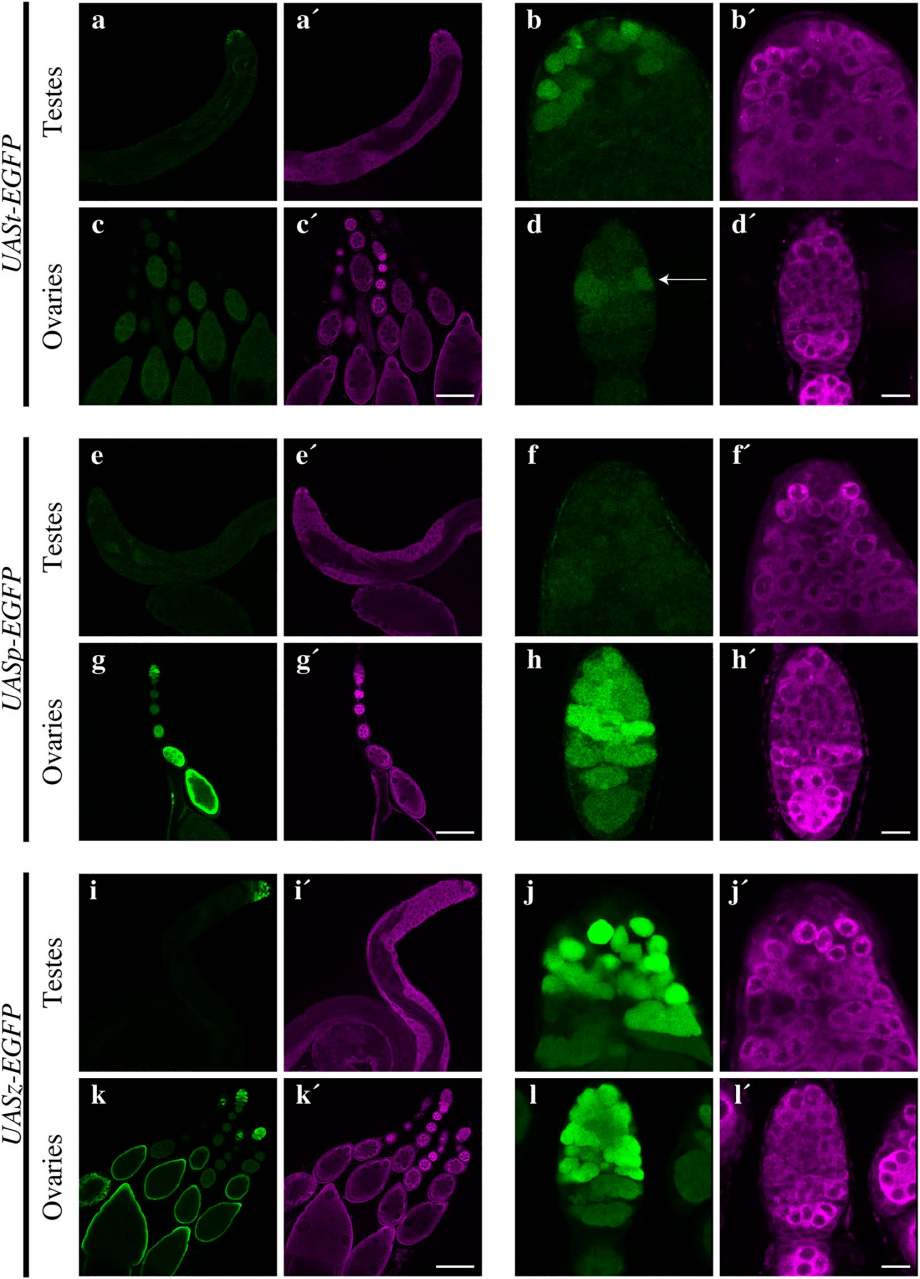
Expression of EGFP from *UASt-*, *UASp-*, and *UASz-EGFP* in the germline cells from testes and ovaries. EGFP expression from (**a**–**d**) *UASt-*, (**e**–**h**) *UASp-*, and (**i**–**l**) *UASz-EGFP* in the germline cells from adult testes (**a,b,e,f,i,j**) and ovaries (**c,d,g,h,k,l**). Low- (**a,c,e,g,i,k**) and high-magnification (**b,d,f,h,j,l**) images are shown. Vasa fluorescence (magenta) indicates germline cells. Testes and ovaries were dissected from adults 4–6 days after eclosion. The adults were derived from females homozygous for *nos-Gal4* mated with *UAS-EGFP* homozygous males. The testes and ovaries were immunostained for EGFP (green) and Vasa (magenta). Signal was obtained using a confocal laser fluorescence microscope with laser intensities and detector settings appropriate for the expression level of EGFP from each *UAS-EGFP* construct at each stage. However, the conditions for signal acquisition were identical for male and female PGCs at each stage. Scale bars: 100 µm (**a, c, e, g, i,** and **k**) or 10 µm (**b,d,f,h,j,l**). Weak expression of EGFP from *UASt-EGFP* was transiently observed in region 1 of the germarium (arrow in **d**). This has been observed previously^6^.

For EGFP and RFP signal detection, fluorescence images of PGCs ranging from the upper surface of the embryos to a depth of 20 µm were obtained using confocal microscopy with an SP5 confocal microscope (Leica Microsystems). For the comparison of EGFP and RFP expression between male and female PGCs, images were obtained on the same day, and image capture was repeated two more times on different days using the same laser intensities, pinhole size, and detector settings. The median values, first quartile values, third quartile values, and P-values (Mann–Whitney *U* test) are shown in Figs. 3 and S2. In Figs. 2, 4, and 5, signal was obtained using a confocal laser fluorescence microscope with laser intensities and detector settings appropriate for the expression levels of EGFP from each *UAS-EGFP* construct at each stage. However, the conditions for signal acquisition were identical for both male and female PGCs at each stage.

EGFP and RFP signal intensities were measured using the Fiji software^24^. Three independent experiments were carried out using different batches of flies.

### Quantification of *UAS-EGFP* mRNA in PGCs

For quantitative RT-PCR (qPCR) analysis of *UAS-EGFP* mRNA, male and female PGCs (100 each) were isolated from embryos at stage 15–16 using fluorescence-activated cell sorting (FACS) as described^22^. cDNAs were synthesized from the sorted PGCs using the Superscript VILO cDNA synthesis Kit (Thermo Fisher Scientific, Cat. No. 11754050). Quantification was performed on a Light Cycler 480 system (Roche) with the QuantiTect SYBR Green PCR Kit (QIAGEN, Cat. No. 204143). Primer pairs used for amplifying *UASt-EGFP*, *UASp-EGFP*, and *UASz-EGFP* mRNA were: UAStEGFP-Fw/UAStEGFP-Rv, UASpEGFP-Fw/UASpEGFP-Rv, and UASzEGFP-Fw/UASzEGFP-Rv, respectively (Table S2). For normalization, *rp49* mRNA was amplified using the primer pair rp49-Fw/rp49-Rv (Table S2).

Data were analyzed using the LightCycler 480 Software (Roche) and Microsoft Excel (Microsoft). Using the ∆∆*C*_T_ method^25^, the values were normalized against *rp49*, and the log_2_FC (male/female PGC) values were calculated. Three independent experiments were performed using independent pools of PGCs.

### Gene Ontology (GO) enrichment analysis

For Gene Ontology (GO) enrichment analysis, we used RNA-seq data obtained from male and female PGCs at stage 15–16^12^. These data have been deposited in DDBJ Bio Project database under Accession No. DRA010934. The raw data were processed using Trimmomatic-0.36^26^ and then aligned to the transcript model of *Drosophila melanogaster* (Flybase; dmel-all-transcript-r6.23.fasta) using Kallisto-0.43.1 with default settings^27^. TPM (transcripts per million) were calculated for each sample. Fold change (log_2_FC) and false discovery rate (FDR) of each transcript were calculated in female PGCs relative to male PGCs using EdgeR^28^. GO enrichment analyses for transcripts twofold enriched in male PGCs relative to female PGCs (log_2_FC > 1 and FDR < 0.01), and vice versa, were performed using DAVID 6.8 (https://david.ncifcrf.gov). ^29, 30^.

### OPP staining

OPP staining was performed using the Click-iT Plus OPP Alexa Fluor 488 Protein Synthesis Assay kit (Thermo Fisher Scientific, Cat#C10456). Embryos were derived from *y w*. Embryos at stage 15–16 were dechorionated in a sodium hypochlorite solution for 10 s. To permeabilize the vitelline membrane, the embryos were incubated in DW-saturated octane for 15 s as described^21^. The permeabilized embryos were incubated for 30 min with gentle shaking in Grace’s Insect Medium (Thermo Fisher Scientific, Cat#11605094) containing 50 µM Click-iT OPP reagent (Thermo Fisher Scientific, Cat#C10459) in the absence or presence of 117 µM cycloheximide. After incubation, the embryos were fixed in 1:1 heptane:fixative for 30 min and devitellinized manually. The fixed embryos were processed for immunostaining for Vasa and Sxl as described above (see Immunostaining**)**. For the Click-iT reaction, the embryos were incubated in the Click-iT reaction cocktail (containing Alexa Fluor 488 picolyl azide) in the dark for 30 min, washed with Click-iT Reaction Rinse Buffer, and washed with PBSTr three times for 20 min each after immunostaining. Samples were mounted in VECTASHIELD Mounting Medium. The sex of each embryo was determined using Sxl staining (Sxl is expressed in the soma in a female-specific manner).

For OPP signal detection, fluorescence images of PGCs ranging from the upper surface of the embryos to a depth of 20 µm were obtained using confocal microscopy with an SP5 confocal microscope. For the comparison of OPP signal intensity between male and female PGCs, images were obtained on the same day, and image capture was repeated two more times on different days using the same laser intensities, pinhole size, and detector settings. OPP signal intensities were measured using the Fiji software^24^. Three independent experiments were carried out using different batches of flies.

### Statistical analysis

Statistical analyses were performed using the R software. Differences were considered to be significant at P < 0.01. Normality of distributions was evaluated by Shapiro–Wilk normality test. Significance of differences was calculated for variables with normal or non-normal distribution by two-sided Student’s t-test or Mann–Whitney *U* test, respectively.

## Results

### Expression of EGFP from UAS vectors in germline cells

Three types of UAS vectors (UASt^2^, UASp^5^, and UASz^6^), which differ in their core-promoter and terminator sequences (Fig. 1), are currently available for use in the Gal4-UAS system. In order to investigate UAS activity, we inserted the *EGFP* gene downstream of the UAS promoters and activated its expression under the control of Gal4 produced from a *nos-Gal4* driver in a germline-specific manner^8^. We examined EGFP protein expression levels in male and female primordial germ cells (PGCs) derived from embryos (Fig. 2). When UASt (Fig. 2a), UASp (Fig. 2b), and UASz (Fig. 2c) were used to express EGFP, protein expression was initially detected in the PGCs of both sexes at stages 7–8, 11–12, and 9–10, respectively (Figure S1). The signal increased to the maximum observed level, which was at stage 15–16 for both sexes (Fig. 2).

### Differences in EGFP expression between male and female PGCs at late embryonic stages

While analyzing EGFP expression levels, we noticed that EGFP expression was higher in the male PGCs in late embryogenesis. The sex difference was first seen at stages 13–14 (UASp) and 9–10 (UASz), and became prominent at the end of embryogenesis (Fig. 2b,c). When EGFP was expressed from UASt, the sex difference was seen at stage 15–16 (Fig. 2a). To further understand this difference, the intensity of the EGFP signal was quantified (Fig. 3). Figure 3 shows that EGFP expression levels varied among the UAS vectors, but male-biased EGFP expression was evident for all three vectors at stage 15–16. Similar male-biased expression was observed at stage 15–16 when RFP was expressed using UASt in PGCs (Figure S2). This suggests that male-biased protein expression is present in PGCs, irrespective of the type of UAS and reporter protein.

We hypothesized that male-biased expression from UAS vectors is evident later in post-embryonic development. We examined EGFP expression in the germline cells of first-, second-, and third-instar larvae and adults (Figs. 4 and 5). In germline cells, EGFP expression levels from all UAS vectors, except for UASt, were male-biased in first- and second-instar larvae but not in third-instar larvae (Fig. 4). Protein expression from UASt was transiently upregulated in the female germline in the first-instar larvae but was male-biased in the second-instar larvae (Fig. 4). In adult gonads, EGFP expression from UASz was detected in the germline cells of both sexes but was not male-biased (Fig. 5i–l). When EGFP was expressed from UASp and UASt, its expression was female- and male-specific, respectively (Fig. 5a–h), except for weak expression from UASt in the germline within region 1 of the ovarian germarium^6^ (Fig. 5d).

### Male-biased EGFP production in PGCs at the post-transcriptional level

**Figure 6:**
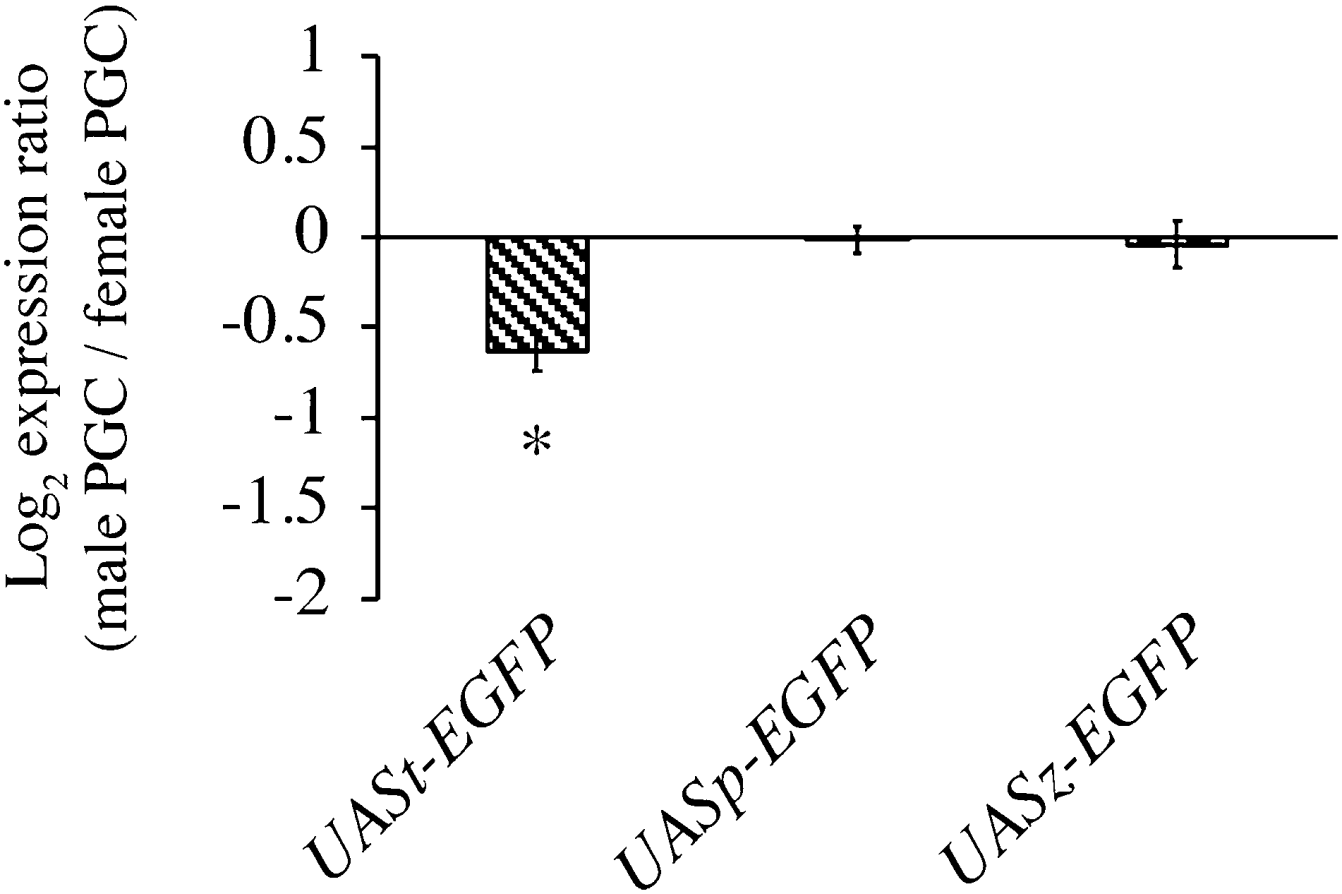
Quantification of *UAS-EGFP* mRNA transcribed from *UASt*-, *UASp*-, and *UASz-EGFP*. Log_2_ expression ratio of transcripts from *UASt*- (left), *UASp-* (middle), and *UASz-EGFP* (right) for male and female PGCs (male/female) at embryonic stage 15–16. We used females homozygous for *nos-Gal4* and *vasa-EGFP* mated with males carrying *UAS-RFP* I on their X-chromosome and homozygous for *UAS-EGFP*. In the embryos derived from these mothers, female PGCs were double-positive for EGFP and RFP and male PGCs were single-positive for EGFP^12, 22^. Quantification of *UAS-EGFP* mRNA in male and female PGCs was performed as described in Materials and Methods. Significance was calculated using the two-sided Student’s t-test. *P < 0.01, male vs. female PGCs.

These observations indicate that EGFP expression is male-biased in the germline cells of late embryos, irrespective of the type of UAS vector used for expression. It is possible that EGFP expression is male-biased due to differences in mRNA expression levels or post-transcriptional regulation. To address this issue, we quantified the amount of *UAS-EGFP* mRNA in the PGCs of both sexes. For this purpose, we used females homozygous for *nos-Gal4* and *vasa-EGFP* mated with males carrying *UASt-RFP* I on their X-chromosome and homozygous for *UAS-EGFP*. In the embryos derived from these mothers, female PGCs were double-positive for EGFP and RFP, and male PGCs were single-positive for EGFP^12, 22^. The male and female PGCs were isolated and processed for analysis of *UAS-EGFP* mRNA levels. To distinguish *UAS-EGFP* mRNA from *vasa-EGFP* mRNA, we used primer pairs complementary to the *EGFP*-coding sequence and the 3´UTR region of the transcript from each *UAS-EGFP* (Fig. 1 and Table S2). We found no difference in the levels of *UAS-EGFP* mRNA transcribed from the UASp or UASz vectors between male and female PGCs (Fig. 6). The mRNA level transcribed from the UASt vector was slightly female-biased but not male-biased (Fig. 6). These observations strongly suggest that the male-biased EGFP expression we observed is due to post-transcriptional regulation of mRNA in PGCs.

### Transcriptome data suggest that translational activity is male-biased in PGCs

Transcriptome data are available for male and female PGCs^12^. To examine whether the transcripts, which are involved in post-transcriptional regulation, are upregulated in male PGCs, we performed Gene Ontology (GO) enrichment analysis for transcripts twofold enriched in male PGCs relative to female PGCs (log_2_FC > 1 and FDR < 0.01) and vice versa. The transcripts with GO terms (biological processes) related to mitosis, such as “DNA replication initiation” and “positive regulation of multicellular organism growth”, were enriched in male PGCs (Table 1). This is presumably because male, but not female, PGCs have just begun cell division at this stage of development^31^. The other GO terms that were significantly enriched in the male-biased transcripts were related to translation, such as “rRNA processing”, “ribosomal large subunit biogenesis”, “ribosome biogenesis”, “maturation of large subunit rRNA”, and “maturation of small subunit rRNA” (Table 1). This strongly suggests that translational activity is male-biased in PGCs.

**Table 1:** Go terms enriched in the transcripts upregulated in male and female PGCs.

GO term†	Description	Count	P-value	FDR
**Upregulated in male PGC**				
*GO:0006364	rRNA processing	17	8.17.E-20	2.97.E-17
GO:0022008	neurogenesis	37	2.05.E-16	3.74.E-14
*GO:0042273	ribosomal large subunit biogenesis	9	6.29.E-12	7.63.E-10
GO:0006260	DNA replication	11	6.18.E-10	5.62.E-08
GO:0009267	cellular response to starvation	13	2.30.E-09	1.68.E-07
*GO:0000470	maturation of LSU-rRNA	7	4.04.E-08	2.45.E-06
*GO:0042254	ribosome biogenesis	8	1.44.E-07	7.48.E-06
GO:0006270	DNA replication initiation	6	6.65.E-06	3.03.E-04
GO:0031120	snRNA pseudouridine synthesis	4	7.84.E-06	3.17.E-04
*GO:0031118	rRNA pseudouridine synthesis	4	1.94.E-05	7.06.E-04
GO:0006261	DNA-dependent DNA replication	5	3.96.E-05	1.31.E-03
*GO:0006360	transcription from RNA polymerase I promoter	4	5.15.E-04	1.45.E-02
*GO:0000462	maturation of SSU-rRNA from tricistronic rRNA transcript (SSU-rRNA, 5.8S rRNA, LSU-rRNA)	5	5.19.E-04	1.45.E-02
GO:0040018	positive regulation of multicellular organism growth	6	6.29.E-04	1.64.E-02
*GO:0000027	ribosomal large subunit assembly	4	1.18.E-03	2.86.E-02
*GO:0017148	negative regulation of translation	5	1.43.E-03	3.25.E-02
*GO:0000154	rRNA modification	3	1.55.E-03	3.31.E-02
GO:0051301	cell division	6	1.76.E-03	3.57.E-02
GO:0007307	eggshell chorion gene amplification	4	2.23.E-03	4.26.E-02
*GO:0000463	maturation of LSU-rRNA from tricistronic rRNA transcript (SSU-rRNA, 5.8S rRNA, LSU-rRNA)	3	4.23.E-03	7.69.E-02
*GO:0000460	maturation of 5.8S rRNA	3	5.39.E-03	9.34.E-02
GO:0030261	chromosome condensation	4	6.25.E-03	1.03.E-01
GO:0010501	RNA secondary structure unwinding	4	6.86.E-03	1.09.E-01
GO:0001522	pseudouridine synthesis	3	9.64.E-03	1.46.E-01
GO:0000381	regulation of alternative mRNA splicing, via spliceosome	5	1.36.E-02	1.98.E-01
GO:0042023	DNA endoreduplication	3	2.36.E-02	3.27.E-01
*GO:0006409	tRNA export from nucleus	2	2.51.E-02	3.27.E-01
GO:0006269	DNA replication, synthesis of RNA primer	2	2.51.E-02	3.27.E-01
GO:0048813	dendrite morphogenesis	7	2.92.E-02	3.67.E-01
GO:0048477	oogenesis	9	4.64.E-02	5.31.E-01
*GO:0006457	protein folding	5	4.79.E-02	5.31.E-01
GO:0006406	mRNA export from nucleus	3	4.84.E-02	5.31.E-01
*GO:0000469	cleavage involved in rRNA processing	2	4.96.E-02	5.31.E-01
*GO:0000466	maturation of 5.8S rRNA from tricistronic rRNA transcript (SSU-rRNA, 5.8S rRNA, LSU-rRNA)	2	4.96.E-02	5.31.E-01
**Upregulated in female PGC**				
GO:0007476	imaginal disc-derived wing morphogenesis	10	1.48.E-03	6.69.E-01
GO:0010506	regulation of autophagy	4	1.14.E-02	1.00.E + 00
GO:0010883	regulation of lipid storage	3	2.26.E-02	1.00.E + 00
GO:0046580	negative regulation of Ras protein signal transduction	3	2.49.E-02	1.00.E + 00
GO:0007409	axonogenesis	4	3.80.E-02	1.00.E + 00
GO:0016255	attachment of GPI anchor to protein	2	4.86.E-02	1.00.E + 00
GO:0030237	female sex determination	2	4.86.E-02	1.00.E + 00
GO:0042742	defense response to bacterium	4	5.00.E-02	1.00.E + 00

^†^GO terms for biological processes that are significantly enriched (P < 0.05, Fisher’s exact test) in the transcripts upregulated in male PGCs relative to the female ones at stage 15–16, and vice versa, are shown. GO enrichment analysis was performed using DAVID ver. 6.8 (https://david.ncifcrf.gov/summary.jsp). The GO terms related to translation are marked with asterisks. The GO terms related to mitosis are underlined.

### OPP staining shows that protein synthesis is higher in male than in female PGCs

**Figure 7:**
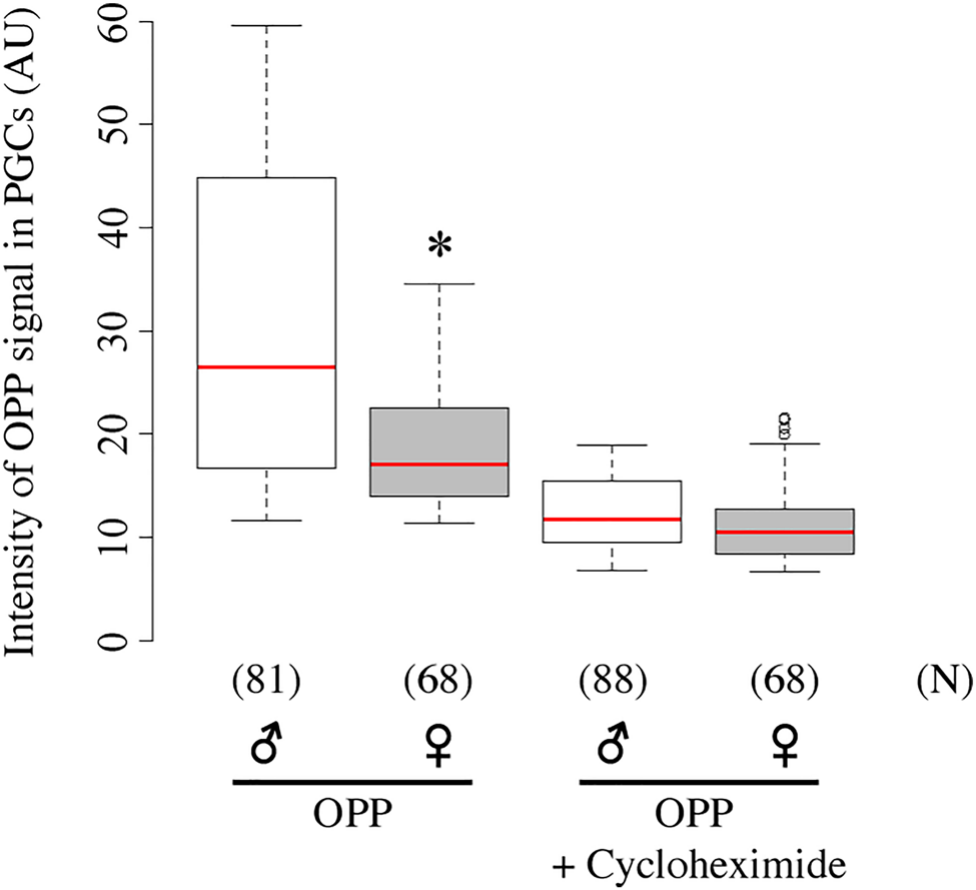
Quantification of OPP signal in PGCs. OPP signal intensity in male (♂, white) and female (♀, grey) PGCs from stage 15–16 embryos, in the absence (left) or presence (right) of cycloheximide. The pixel intensity for OPP signal is shown. Pixel intensities were obtained using a confocal laser fluorescence microscope with fixed laser intensities and detector settings (i.e., the settings did not vary with OPP signal intensity). ﻿Each box plot represents median values (red bars) and first (25%) and third (75%) quartile values. Whiskers extend 1.5 times the interquartile range (IQR) from the 25% and 75% quartile. The upper and lower whisker indicate the largest and smallest value that are no greater and lower than 75% plus 1.5 IQR and 25% minus 1.5 IQR, respectively. White circles represent outliers. Significance was calculated using the Mann–Whitney *U* test. *P < 0.01, male vs. female PGCs. The number of PGCs (N) examined is indicated in parentheses. AU: arbitrary units.

To determine whether translational activity was male-biased in PGCs, we quantified protein synthesis in male and female PGCs from late embryos using O-propargyl-puromycin (OPP) staining. OPP, an alkyne analog of puromycin, is incorporated into nascent polypeptide chains during polypeptide elongation on ribosomes, and its incorporation into the nascent polypeptide chains blocks protein synthesis^32^. Incorporated OPP can be visualized by Click reaction between a fluorescent azide and OPP^32^. Thus, OPP staining is a sensitive method for detecting protein synthesis. We found that OPP signal was higher in male than in female PGCs (Fig. 7). By contrast, when the embryos were treated with cycloheximide, which blocks translational initiation, the OPP signal was decreased in PGCs of both sexes, and no difference in OPP signal intensity was detected between male and female PGCs (Fig. 7). These observations show that protein synthesis is higher in male than in female PGCs.

## Discussion

In this study, we found that the UASt, UASp, and UASz vectors were all active under the control of the *nos-Gal4* driver in the germline cells of embryos and larval gonads (Figs. 2 and 4), although the EGFP expression levels were significantly lower in the germline when we used UASp (Fig. 3). Therefore, our data indicate that the UASt and UASz vectors function more effectively in the germline at the embryonic and larval stages than UASp. By contrast, in the germline stem cells and their descendants in the adult ovaries, EGFP expression from UASt was barely detectable, whereas expression from UASz and UASp was observed at high and moderate levels, respectively (Fig. 5c,d,g,h,k,l). Similar observations have been reported previously^2, 5, 6^. Conversely, in adult testes, EGFP expression from UASt was observed in the germline cells from the distal tip region of the testes (Fig. 5a,b), but expression from UASp was undetectable in the testes (Fig. 5e,f). When we used UASz, the level of EGFP expression was higher than when we used UASt (Fig. 5a,b,i,j). Because the sequence targeted by *Hsp70*-directed Piwi-interacting RNAs (pi-RNAs) was deleted from UASt to generate UASz^6^, this observation suggests that these piRNAs are present in the distal tip region of the testes. Taken together, these data demonstrate that all three UAS vectors can be used to express genes in the germline of embryos and the larval gonads of both sexes, but UASz alone is useful for gene expression in the germline of adult ovaries and testes.

Moreover, we observed male-biased protein expression in the PGCs from late embryos, irrespective of the vector used for expression or the reporter protein. The male-biased expression of EGFP from *UASp-* and *UASz-EGFP* was also observed in the germline cells from first-instar larvae, and expression from all *UAS-EGFP* vectors was male-biased in second-instar larvae. The male-biased EGFP expression may be due to differences in translation or protein degradation between male and female PGCs. GO enrichment analyses revealed that the genes with translation-related GO terms exhibited male-biased expression in PGCs (Table 1). Furthermore, OPP staining shows that protein synthesis was male-biased (Fig. 7). Based on these findings, we conclude that translational activity is higher in male than in female PGCs, resulting in male-biased expression of EGFP protein from UAS vectors.

In *Drosophila*, the sexual identity of the germline cells is regulated by cell-autonomous and non-cell-autonomous cues and is thought to be determined in late embryogenesis^15, 16, 31^. Hence, it is possible that male-biased translation activity may involve sexual differentiation of the germline cells. However, the functional importance of this male-biased upregulation of translation in germline development remains elusive. In the future, combining translational repression in male PGCs with upregulation of translation in female PGCs could result in a powerful approach to clarify the biological significance of male-biased translation in PGCs.

## Supplementary Information

Supplementary Information.

## Data Availability

All data and materials produced in this study are available from the corresponding authors upon reasonable request.
