# Supplementary material for: Male-biased protein expression in primordial germ cells, identified through a comparative study of UAS vectors in Drosophila
Source: Sci Rep. 2021 Nov 2;11:21482. doi: 10.1038/s41598-021-00729-1 (PMC8564522; doi:10.1038/s41598-021-00729-1)
Supplement: Supplementary file 1 — Supplementary Information. [file 41598_2021_729_MOESM1_ESM.pdf]

Male-biased protein expression in primordial germ cells, identified through a comparative study of UAS vectors in *Drosophila*

Masaki Masukawa[1,2], Yuki Ishizaki[2,3], Hiroki Miura[2,3], Makoto Hayashi[1,2,3], Ryoma Ota[4,5] and Satoru Kobayashi[1,2,3*]

[1] Degree Programs in Life and Earth Sciences, Graduate School of Science and Technology, University of Tsukuba, Tsukuba, Ibaraki, 305-8577, Japan

[2] Life Science Center for Survival Dynamics, Tsukuba Advanced Research Alliance (TARA), University of Tsukuba, Tsukuba, Ibaraki, 305-8577, Japan

[3] Graduate School of Life and Environmental Sciences, University of Tsukuba, Tsukuba, Ibaraki, 305-8577, Japan

[4] Department of Biosciences, Faculty of Science and Engineering, Teikyo University, Utsunomiya, Tochigi, 320-8551, Japan

[5] Division of Integrated Science and Engineering, Graduate School of Science and Engineering, Teikyo University, Utsunomiya, Tochigi, 320-8551, Japan

[*]Corresponding author:

Satoru Kobayashi; skob@tara.tsukuba.ac.jp, Tel: +81- 029-853-5881

Supplementary Table S1.

Primers used for the construction of *UASt-* and *UASz-EGFP.*

Primer name	Primer sequence (5´–3´)†
UASt-KpnI-EGFP-Fw	GGCCGCGGCTCGAGggtaccATGGTGAGCAAGGGCGAGGAGC
UASt-KpnI-EGFP-Rv	AAAGATCCTCTAGAggtaccTTACTTGTACAGCTCGTCCATGCCG
UASz-KpnI-EGFP-Fw	AAGGATCCCTCGAGggtaccATGGTGAGCAAGGGCGAGGAGC
UASz-KpnI-EGFP-Rv	ATTCTAGAACTAGTggtaccTTACTTGTACAGCTCGTCCATGCCG

†Lowercase letters indicate the sequence recognized by *Kpn*I. The overlapping ends for cloning (In-fusion HD kit) are underlined.

Supplementary Table S2.

Primers used for the quantification of *UAS-EGFP* mRNA.

Primer name	Primer sequence (5´–3´)
UASt-EGFP-Fw	ACATGGTCCTGCTGGAGTTC
UASt-EGFP-Rv	TTTGTCCAATTATGTCACACCAC
UASp-EGFP-Fw	ATCACTCTCGGCATGGAC
UASp-EGFP-Rv	TGTGTTCTCAACTTCAAAGGC
UASz-EGFP-Fw	GGATCACTCTCGGCATGGAC
UASz-EGFP-Rv	TCGTAATCTTCCTAATGATCACATTTT
rp49-Fw	AGTCGGATCGATATGCTAAGCTG
rp49-Rv	ATGTTGGGCATCAGATACTGTCC

Supplementary Figure S1.

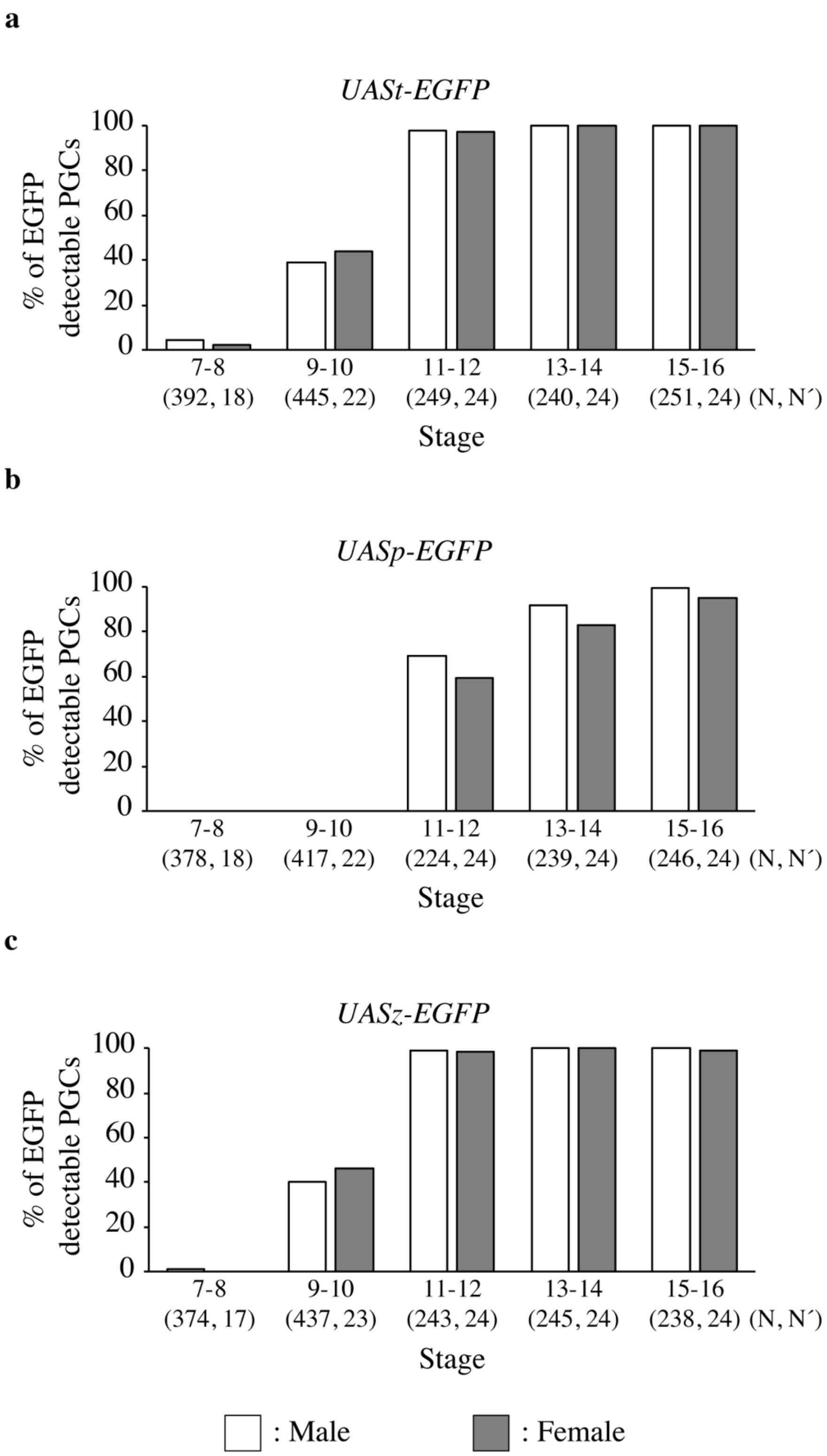

Figure S1.

The percentages of male and female PGCs expressing EGFP from *UASt-*, *UASp-*, and *UASz-EGFP.*

The percentage of male (white bars) and female (grey bars) PGCs expressing EGFP from (a) *UASt-*, (b) *UASp-*, and (c) *UASz-EGFP* at embryonic stages 7–8, 9–10, 11–12, 13–14, and 15–16. Embryos, derived from females homozygous for *nos-Gal4* mated with *UAS-EGFP* homozygous males, were immunostained for EGFP, Vasa, and Sxl (expressed in the soma in a female-specific manner). The numbers of PGCs (N) and embryos (N´) examined are indicated in parentheses.

Supplementary Figure S2.

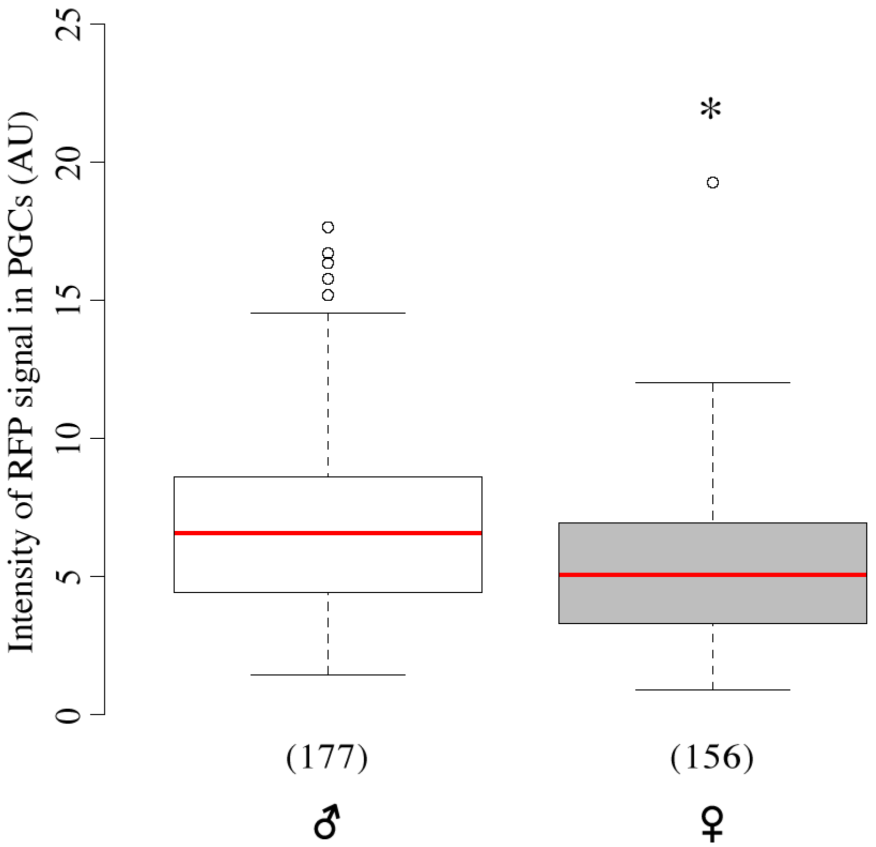

Figure S2.

Quantification of RFP expressed from *UASt-RFP* III in PGCs.

Expression level of RFP expressed from *UASt-RFP* III in male (♂, white) and female PGCs (♀, grey) from stage-15–16 embryos. The pixel intensity for the RFP signal is shown. Pixel intensities were obtained using a confocal laser fluorescence microscope with the same laser intensities and detector settings. Each box plot represents median values (red bars) and first (25%) and third (75%) quartile values. Whiskers extend 1.5 times the interquartile range (IQR) from the 25% and 75% quartile. The upper and lower whisker indicate the largest and smallest value that are no greater and lower than 75% + 1.5 IQR and 25% - 1.5 IQR, respectively. White circles represent outliers. Significance was calculated using the Mann-Whitney *U* test. *P < 0.01, male vs. female PGCs. The number of PGCs (N) examined is indicated in parentheses. AU: arbitrary unit.
